# Pharmacodynamic Effects of an Angiotensin II Receptor-Antagonist in Phase I—Comparison between Healthy Subjects and Patients with Hypertension

**Published:** 2007-03-22

**Authors:** Georg Wensing, Klaus Ochmann, Michael Boettcher, Anja Schäfer, Jochen Kuhlmann

**Affiliations:** Institute of Clinical Pharmacology, Bayer HealthCare AG, Germany

## Abstract

Biomarkers are increasingly used to provide decision making data early in phase I by showing Proof of Mechanism or Proof of Concept (PoM/PoC). For antihypertensive agents, the administration of multiple doses (md) to hypertensive patients is assumed to be necessary for an early go/no-go decision. We compared the effects of an Angiotensin II receptor antagonist (ARA) on Plasma Renin and blood pressure (BP) following an oral single dose (sd) and once daily md for seven days to healthy volunteers and patients with essential hypertension (diastolic BP 95 mmHg to 114 mmHg; systolic BP 130 mmHg to 200 mmHg). Methods: 5–12 healthy male subjects/dose received 10 mg to 300 mg ARA sd and 50 to 300 mg md for 7 days; patients (9–10/dose) received 20 mg–400 mg ARA for 7 days. The studies were designed as randomized, single-blind, placebo-controlled, group comparison or crossover dose-escalation studies. Plasma Renin and BP were monitored up to 24 hours after dosing. Results: Plasma Renin showed a high interindividual variability in both healthy volunteers and patients. Healthy subjects showed a dose- and time-related increase in plasma Renin after sd from 40 mg to 300 mg and md of 50 mg to 300 mg (p < 0.05 for doses of 200 mg and 300 mg). In patients, increases in plasma Renin occurred at 8 hours and beyond starting at sd of 100 mg and md of 50 mg (p < 0.05 for the dose of 400 mg). While healthy volunteers showed no relevant decrease in BP, in hypertensive patients a reduction in BP in doses of 100 mg to 400 mg occurred (p < 0.05); effects were more pronounced after md compared to sd. Conclusion: Early PoM for an antihypertensive agent can be shown by use of laboratory biomarkers following sd to healthy subjects. PoC can be achieved after sd in hypertensive patients. Administration of sd to healthy volunteers is sufficient for an early go/no-decision.

## Introduction

Drug development has faced a change in paradigm in recent years. Due to increasing development costs and time (Grabowski et al. 2002; DiMassi, 2002) in combination with a reduced number of drug approvals (Frantz and Smith, 2003; The CMR International, 2004), the Factbook, 2004) the pharmaceutical industry seeks to reduce attrition rates by providing decision-making data early in phase I and phase IIa (Kuhlmann, 1999; Colburn, 2000; Grabowski et al. 2002; DiMassi, 2002; Frantz and Smith, 2003). While pharmacokinetics appears to be no longer a major cause of failure (Prentis et al. 1988; Frank and Hargreaves, 2003; Walker, 2004), there are still high failure rates due to lack of safety and efficacy especially in late stages of development. In addition, after marketing, drug treatment suffers from a high number of poor responders even to commonly used drugs and an increasing rate of withdrawals due to adverse events (Silber, 2001). Thus, the challenge is to provide reliable data which allow predicting for the efficacy or the safety of a drug early in clinical development. As clinical endpoints or true surrogates which predict for a clinical endpoint can usually not be applied to early short-term phase I or phase IIa studies, a real proof of principle can only be achieved in exceptional cases. Therefore, biomarkers are increasingly used to assess the potential of a drug for success in later stages of development.

A biomarker of drug effects should ideally reflect a process on the critical path between the pharmacological action of the drug and its effect on a disease. The most common definition of a biomarker is given by the National Institute of Health Biomarkers Definition Working Group (Biomarkers Definition Working Group, 2001). They define a biomarker as a laboratory measurement or physiological sign in association with a physiologic process of putative therapeutic or diagnostic value. A new mechanistic classification of 7 types of biomarkers is proposed by Danhof et al. based on the location of the biomarker in the chain of events from underlying subject genotype or phenotype via drug/metabolite plasma concentrations, molecular target occupancy, molecular target activation, physiological measures, pathological measures/disease processes through to clinical scales (Danhof et al. 2005). Thus, biomarkers vary with respect to their closeness to the intended therapeutic response. Although biomarkers are in general not used for regulatory purposes and therefore need not to be fully validated against clinical endpoints, company decisions to proceed or discontinue a project will rely on the biomarker results. Thus, the selection of the optimal biomarker as well as the duration of treatment and the selection of the study population are critical.

Biomarkers may have their greatest benefit in providing early Proof of Mechanism (PoM) or Proof of Concept (PoC) in exploratory drug development in man. The terms PoM, PoC and Proof of Principle (PoP) are inconsistently used throughout the literature. We define PoM as proof that the proposed pharmacological mechanism of a drug is valid in man, e.g. a drug binds to a receptor or inhibits an enzyme (Kuhlmann and Wensing, 2006). The differentiation between PoC and PoP is less clear, and often both terms are used synonymously. If one tries to differentiate, the term PoC could be used as the translation of the pharmacological effect into a meaningful laboratory or clinical biomarker in healthy subjects or patients, whereas PoP could be restricted to effects on a valid clinical surrogate or at least a candidate surrogate in the target population (Kuhlmann and Wensing, 2006).

For antihypertensive agents, especially those directly acting on the Renin-Angiotensin-System, the activity of vasoconstrictive hormones and the drop in blood pressure may serve to show an early PoM and PoC, respectively. In healthy volunteers, the vasodilative effect of an antihypertensive agent is usually counter-regulated by a compensatory increase in sympathetic nerve activity or vasoconstrictive hormones as Renin or Angiotensin. Therefore, a substantial decrease in blood pressure is often not observed at potentially therapeutic doses. Even if blood pressure reductions are observed, a meaningful dose response curve can usually not be established as a further drop in blood pressure is often poorly tolerated. However, the activity of vasoconstrictive hormones as Renin and Angiotensin may be used as a sensitive biomarker for an early PoM and the demonstration of a dose-effect relationship in healthy volunteers. To observe meaningful blood pressure lowering effects and to achieve PoC, the administration of multiple doses (md) to hypertensive patients is assumed to be necessary; however, the duration of treatment is unclear.

BAY 10-6734 is an investigational orally active dihydropyridine derivate with a selective competitive antagonism at the Angiotensin II AT1 receptor subtype developed for the treatment of arterial hypertension. Its main active metabolite BAY 10-6735 is a reversible Angiotensin II AT1 receptor antagonist which in-vitro has been shown to be more effective than BAY 10-6734 but is poorly absorbed from the gastrointestinal tract (Knorr et al. 1996). Although the administration of BAY 10-6734 to healthy volunteers is safe and well tolerated and the compound shows favorable pharmacokinetic and pharmacodynamic properties (Breithaupt-Grögler et al. 1997), the development of the compound was not further pursued for internal reasons. We used single dose (sd) and md studies of BAY 10-6734 to compare the pharmacodynamic effects of different doses of an anti-hypertensive agent in healthy volunteers and patients with essential hypertension. The purpose of the investigation was to evaluate if decision making data can be provided in a phase I setting by sd or md studies in healthy volunteers or if studies in hypertensive patients are necessary. In addition, the safety of the compound in PoM/PoC studies in healthy volunteers compared to patients was evaluated.

## Materials and Methods

### Study design

#### Healthy volunteer studies

The healthy volunteer studies were conducted at the study ward of the Institute of Clinical Pharmacology of Bayer HealthCare AG in Wuppertal, Germany. The study protocols were approved by the Ethics Committees of the North Rhine Medical Council, Düsseldorf, Germany, and the studies were conducted in accordance with the Declaration of Helsinki, Good Clinical Practice guidelines and German drug law. All subjects gave written informed consent to participate in the studies. Healthy Caucasian male subjects, aged 18–45 years, were eligible to participate.

The healthy volunteer studies were first in man dose-escalation studies to evaluate the safety, tolerability and pharmacokinetics of the compound. Thus, no volunteer was allowed to participate in more than one dose step. Doses were escalated as long as no unacceptable side-effects occurred. As both studies had pilot character, no efforts were made to complete the groups up to the planned sample size if some volunteers did not qualify for participation at screening and a minimum number of eight subjects on active medication (n = 5 for dose steps with a planned sample size of six subjects on active treatment) for the evaluation of safety, tolerability and blood pressure was included. The 10 mg dose was the dose released as first in man dose, thus, a lower number of volunteers was included in this dose step. As part of the exploratory nature of a first in man study, the first dose steps were performed with a solution to allow for a better dose adjustment as long as the dose to be used in later studies were unknown. At higher doses, a capsule was used.

The sd study was performed in a randomized, double-blind, placebo-controlled two-fold crossover design (12 planned subjects/dose; n = 6 for the 10 mg dose). Subjects were randomly allocated to receive doses of 10 mg, 20 mg, 40 mg and 80 mg as oral solution and 200 mg and 300 mg as capsule. The md study was randomized, double-blind, placebo-controlled with 8 (50 mg and 100 mg) and 12 (200 mg and 300 mg) planned subjects/dose. For doses of 50 mg and 100 mg, a parallel group design was chosen (n = 6 verum/2 placebo), doses of 200 mg and 300 mg were administered in a crossover design. The higher starting dose and the different doses in the md study were selected on the basis of the favourable safety and tolerability in sd studies in healthy volunteers as well as the availability of capsules. All doses were given once daily as capsule in the morning for 7 days.

On the study days (day 1 and 7 of treatment = days of first and last administration of study drug), study drugs were administered with 150 ml of water at 8 am after 10 hours overnight fast with breakfast being allowed 2 hours after drug administration, on the other days, study drugs were taken with breakfast. The wash-out phase between the crossover steps was at least 7 days. A full medical examination was performed at follow-up, no more than 7 days after the last study dose. Each BAY 10-6734 dose step was initiated when the results of the previous dose step were available and if there were no unacceptable adverse effects.

#### Patient study

The study in hypertensive patients was conducted at LAB GmbH Neu-Ulm, Germany. The study protocol was approved by the local Ethics Committee and the study was performed in accordance with the Declaration of Helsinki, Good Clinical Practice guidelines and German drug law. All subjects gave written informed consent to participate in the studies. Male Caucasian patients, aged 35–70 years, with established essential hypertension (diastolic blood pressure (DBP) between 95 mmHg and 114 mmHg and systolic blood pressure (SBP) above 130 mmHg and below or equal to 200 mmHg) were eligible to participate. Hypertension was evident for at least six months. Patients unresponsive to Angiotensin Converting Enzyme inhibitors as well as patients with secondary hypertension or clinically relevant diseases of other organs (cardiovascular, central-nervous system, gastrointestinal and hepatic, pulmonary, renal, endocrine) including insulin dependent or uncontrolled diabetes were not eligible for participation. All laboratory values including parameters for renal function had to be normal before start of the study.

The study was designed as a randomized, double-blind, placebo-controlled, group comparison, dose-escalation study. Again, doses were escalated as long as no unacceptable side effects occurred. Based on the unknown sensitivity of hypertensive patients to the blood pressure lowering effect of the compound, a lower starting dose was selected in the patient study than in the healthy volunteer study. Hypertensive patients received 20 mg, 50 mg, 100 mg, 200 mg, 300 mg and 400 mg as capsules once daily for 7 days. A placebo run-in period of three to six weeks was followed by 13–16 days of hospitalization. Two days before administration of the study drugs, patients could adapt to the study facilities (placebo treatment). For safety reasons, the administration of the 400 mg dose was preceded by three days of treatment with 200 mg.

Study drug administration, follow-up investigation and dose escalation procedure were comparable to healthy volunteer studies.

### Safety and tolerability

Safety and tolerability were monitored at predefined intervals by non-leading questioning (occurrence and severity of adverse events), laboratory parameters and vital signs (SBP, DBP, heart rate and electrocardiogram (ECG)) daily up to 72 hours after the last drug administration.

### Biomarkers

DBP was taken as the primary clinical biomarker. The vasoconstrictive hormone Renin served as the primary laboratory biomarker. In addition, measurements of Angiotensin II and Aldosterone were performed. Vasoconstrictive hormones were determined by commercially available radioimmuno assays (Dia Sorin: Plasma-Renin Activity; Bühlmann Laboratories AG: Angiotensin II; DPC Biermann: Aldosterone) at the Institute of Clinical Pharmacology, Bayer HealthCare AG, Wuppertal. To note, the assay for Renin changed from the healthy volunteer studies to the patient studies. Thus, Renin is given as Renin activity (RA; μg/l/h) in the healthy volunteer studies and Renin concentration (RC; μU/ml) in the patient study, respectively. Blood pressure and vasoconstrictive hormones were determined in supine position after 15 minutes of rest on days 1 and 7 at 2, 4, 6, 8, 12, 16 and 24 hours after drug intake in the sd healthy volunteer study and the md patient study. In the md study in healthy volunteers, hormones were determined at baseline and at 2, 4, 12 and 24 hours after drug administration.

### Pharmacokinetics

Plasma samples for the determination of BAY 10-6734 and BAY 10-6735 were taken in timed intervals on day 1 and 7 up to 72 hours after the last dose. Plasma concentrations were determined by HPLC with on column focusing and fluorescence detection at the Institute of Clinical Pharmacology, Bayer HealthCare AG, Wuppertal. Pharmacokinetic parameters were evaluated using non-compartmental methods.

### Statistics

All data were tabulated and subjected to a descriptive statistical evaluation. The statistical analysis was based on a linear mixed model for the analysis of repeated measurement design. The parameter of interest was measured repeatedly within each treatment at different time points. Beside the overall mean, fixed effects were assumed for the classification variable “treatment and time.” To allow different time courses, the interaction terms between treatment and time were added to the model. As variance components, the random effects due to subject variability and measurement errors were considered in the model. Based on these models, means for the treatment groups (least square means) as well as treatment differences and the corresponding p values for testing of no group differences were derived. In addition, data of different time points were compared to baseline by using the student’s t-test. P < 0.05 was used as the minimum level of significance. Statistical evaluation was performed using the SAS software package Version 8.

## Results

### Study population

Study characteristics including study designs and number of subjects enrolled are given in Table 1. Healthy volunteers participating in sd and md studies were aged 20 to 45 years, their body weights ranged from 53 to 110 kg. No significant differences existed between the different treatment groups with respect to age and body weight. Based on the natural occurrence of hypertensive disease, the age range for inclusion of hypertensive patients was different from healthy volunteers. Thus, patients with hypertension were older than healthy subjects and also showed a higher body weight. Their age ranged from 36 to 70 years, the body weights from 60–108 kg. Again, no significant differences existed between the different treatment groups with respect to age and body weight. Due to the rigid exclusion criteria, patients with hypertension showed no other signs of relevant organ dysfunction and, therefore, no additional differences to healthy volunteers.

### Laboratory biomarkers

**Figure 1:**
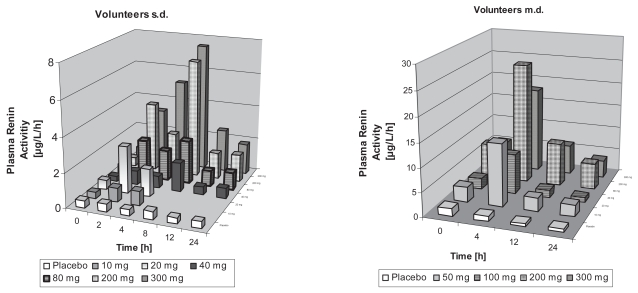
Plasma Renin activities in healthy volunteers at different times 1 (sd) and 7 (md) days after administration of various doses of BAY 10-6734.

In healthy volunteers, there was a wide interindividual variability in increase in RA in plasma for all doses. Overall, time- and dose-related increases in mean RA were observed after sd from 10 mg to 300 mg (Figure 1). Maximum effects in mean RA occurred at 4 h after drug administration for doses of 10 mg to 20 mg and at 8 h after drug administration for doses from 40 mg to 300 mg. Higher than 2 fold elevations compared to baseline were still present at 24 hours for doses from 80 mg to 300 mg. Statistical analysis showed statistically significant differences within the 200 mg and 300 mg dose groups (p < 0.05). For both doses values at all time points were significantly different from baseline (p < 0.05). When maximum values were compared to baseline irrespective of the time point they occurred (Table 2), slight increases in RA were observed for doses of 10 mg to 40 mg (p < 0.05 for the 10 mg dose) while a strong and dose-dependent increase was observed for doses of 80 mg (p < 0.01; 6.5 fold), 200 mg (p < 0.01; 14.8 fold) and 300 mg (p < 0.001; 19 fold). In the md study, baseline values on day 1 were comparable to the data in the sd study (no statistically significant difference; Table 3). On day 7, RA before drug administration was significantly higher than on day 1 (Figure 1; p < 0.05 for doses of 100 mg, 200 mg, and 300 mg). Maximum changes in mean RA occurred at 4 hours after drug administration. Higher than 2 fold elevations were present at 24 hours for doses equal to or higher than 100 mg compared to baseline before the first dose. Again, statistical analyses revealed statistically significant differences within the 200 mg and 300 mg dose groups (p < 0.05) with all time points being significantly different from baseline on day 1 (p < 0.05). If maximum values were compared to baseline irrespective of the time point they occurred, increases in RA were more pronounced on day 7 compared to day 1 or sd (Table 3). To note, as no 8 hour values were taken, the magnitude of increase may be underestimated when compared to the sd healthy volunteer study. Angiotensin II and Aldosterone levels showed a high variability and, especially for Aldosterone, a high number of values below LLOQ (lower limit of quantification). Due to this variability, no significant changes were observed. However, if maximum changes of Angiotensin II were compared to baseline irrespective of the time point they occurred, a significant increase was observed for all doses except the 100 mg dose on day 1 (Table 3). Changes for the 200 mg and 300 mg dose were in the order of magnitude as observed for RA. Measurements of Aldosterone did not result in any statistically significant difference.

**Figure 2:**
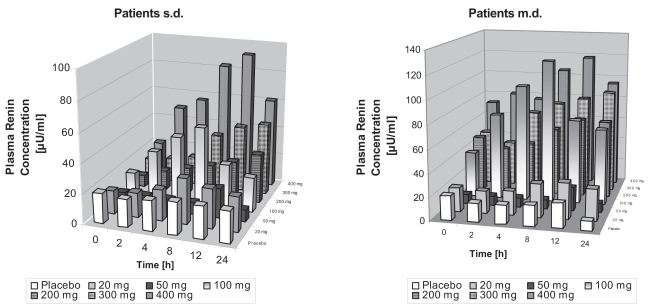
Plasma Renin concentrations in patients with essential hypertension at different times 1 (sd) and 7 (md) days after administration of various doses of BAY 10-6734.

In hypertensive patients, there was a comparable intraindividual variability in RC in plasma. Again, time- and dose-related increases in mean RC occurred after single doses from 100 mg to 400 mg (Figure 2). Maximum effects were observed at 8–12 h after drug administration. Higher than 2fold elevations compared to baseline were still present at 24 hours for doses from 100 mg to 400 mg. Due to the great interindividual variability, a statistically significant increase was only detected for the 400 mg dose (p < 0.05), with all time-points being significantly different from baseline (p < 0.05). As in healthy volunteers, following md, baseline values on day 7 were significantly higher than on day 1 for doses of 50 mg and above (p < 0.05). At 24 h, higher than 2 fold elevations were present for doses from 100 mg to 400 mg. Again, a statistically significant difference was only observed for the 400 mg dose group (p < 0.05). Thereby, all increases at 8 hours after drug administration and beyond showed a statistically significant difference form baseline (p < 0.05). If maximum values on days 1 and 7 irrespective of the time point they occurred were compared to baseline, significant increases were observed for all but the 100 mg dose (Table 3). Due to the higher baseline values, the magnitude of increase was lower than observed for healthy volunteers (maximum change about eight fold). Again, Angiotensin II and Aldosterone levels showed a high variability and, especially for Aldosterone, a high number of values below LLOQ. As in healthy volunteers, statistically significant differences for maximum changes of Angiotensin II compared to baseline were observed for all doses except the 100 mg dose (p < 0.05; Table 3). No difference for Aldosterone was observed.

## Clinical Biomarkers

**Figure 3:**
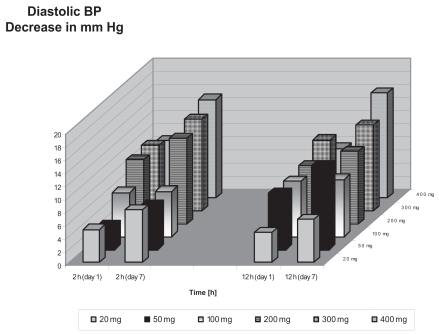
Diastolic blood pressure reductions in patients with essential hypertension at different times 1 (sd) and 7 (md) days after administration of various doses of BAY 10-6734.

In healthy volunteers, a slight decrease in DBP occurred only with the 300 mg dose for sd and md (statistically not significant). In contrast, hypertensive patients showed a statistically significant reduction in DBP in doses of 100 mg to 400 mg at 2 hours and 50 mg to 400 mg at 12 hours after drug administration (p < 0.05; Figure 3). No clear dose response relationship was observed. Effects were more pronounced after md compared to sd (Figure 3). The mean maximum decrease in DBP was −16 mm Hg for the 400 mg dose on day 7–12 h after dosing. Normalization of DBP was observed in some patients on 300 mg and 400 mg after 7 days of treatment. Screening values (values before start of the washout period), baseline values and values at the end of treatment for the different doses are given in Table 4. Relevant effects were still observed 24 hours after the last dose.

## Pharmacokinetics/Pharmacodynamics

**Figure 4:**
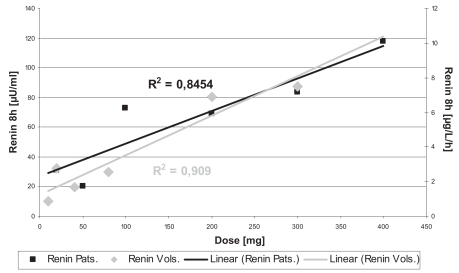
Relationship between mean Plasma Renin activities and concentrations and doses of BAY 10-6734 in healthy volunteers and patients with essential hypertension on day 1 eight hours after dosing.

**Figure 5:**
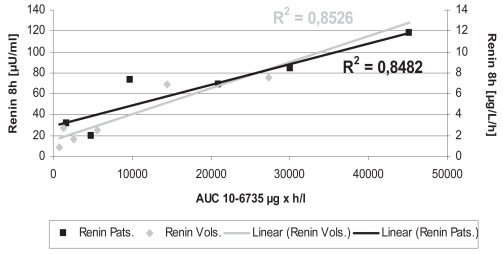
Relationship between mean Plasma Renin activities and concentration and AUC of BAY 10-6735 in healthy volunteers and patients with essential hypertension day 1 eight hours after dosing.

The pharmacokinetic parameters of BAY 10-6734 and BAY 10-6735 following single doses in healthy volunteers are given in Table 5. In healthy volunteers, dose-dependent increases in plasma concentrations for BAY 10-6734 and BAY 10-6735 were observed. No differences were observed between day 1 and day 7 of multiple dose treatment. Pharmacokinetics in patients with hypertension did not differ from healthy volunteers. Doses of BAY 10-6734 and AUC (area under the curve) of BAY 10-6735 significantly correlated to RA and RC in healthy volunteers and patients, respectively (Figures 4 and 5). No clear relationship to DBP reductions in patients was observed.

## Safety

BAY 10-6734 was safe and well tolerated when given to healthy volunteers and patients with essential hypertension. The adverse event pattern was comparable to what is usually observed in healthy volunteer studies in phase I and mostly comprised adverse events like headache or nausea irrespective of active drug or placebo. The only drug related adverse events related to the mode of action were two orthostatic reactions—one in the highest dose in a patient with essential hypertension which resulted in discontinuation of the study drug and one in a healthy volunteer on the 300 mg dose, although this event occurred under both active drug and placebo. Due to the observed slight blood pressure reduction and the orthostatic reaction at the 300 mg dose in healthy volunteers and the orthostatic reaction in one patient at the 400 mg dose, these doses were taken as the maximum tolerated dose for healthy volunteers and patients, respectively, and no further dose escalation was performed.

## Discussion

The results of the present studies demonstrate that the determination of a laboratory biomarker such as Renin allows achieving an early PoM following a single dose of an Angiotensin II receptor-antagonist in healthy volunteers. Multiple dosing in healthy volunteers and single or multiple dosing in patients do not add any further information, neither with respect to a go/no-go decision nor with respect to dose selection: despite a high interindividual variability, dose-effect relationships for Renin were comparable for healthy volunteers and patients pointing at the 300 mg and 400 mg doses to be used in phase II and III. Probably due to the absence of influence of disease, Renin changes in healthy volunteers were even higher than in patients and also showed a better dose-response relationship. The Renin assay changed from the healthy volunteer studies to the patient study and Renin activity to Renin concentration. In a study in healthy volunteers and diabetic patients, a strong correlation between Renin activity and Renin concentration was demonstrated for both study populations (Valabhji J et al. 2001). Thus, data for healthy volunteers and patients in the present study should be comparable.

In contrast to the results for Renin, no relevant effects on blood pressure were detectable in healthy volunteers following single or multiple dosing in the investigated dose range, thus PoC can only be achieved in hypertensive patients. Single dosing in patients allows an early PoC, as relevant reductions in blood pressure could be observed but multiple dosing in patients for seven days adds certainty about the dose-effect relationship, the potency and the duration of action of the compound. However, a short treatment period of 7 days does not allow the assessment of the maximum effect of the compound as it is not clear if steady state in reduction of blood pressure was achieved. Thus, the advantage of 7 days versus a 1 day study in patients is questionable. Instead, a longer treatment period in comparison to a gold standard might be preferable.

An alternative approach to achieve early PoM/PoC is the application of provocation studies in healthy volunteers as often applied in the development of antiasthmatics (Smith et al. 1985; Wensing et al. 1996). The pharmacodynamic properties of an Angiotensin II receptor antagonist can also be quantitatively characterized by provocation testing from the rightward shift of the agonist dose-response curve after intravenous administration of Angiotensin II to healthy volunteers. For BAY 10-6734, such a study was performed in healthy volunteers applying single oral doses of 20 to 300 mg (Breithaupt-Grögler et al. 1997). In this study, BAY 10-6734 showed to be a fast-acting, highly potent and orally active Angiotensin II antagonist in man. The observed effects were dose dependent and clearly suggested doses of 300 mg and 400 mg as clinically effective doses which might allow maintaining a relevant blood pressure lowering effect for 24 hours. These results correspond to the findings of dose escalation studies in healthy volunteers. Although provocation studies in general are complicated and more time consuming as dose escalation studies, they offer additional value for an early go/no-go decision as they allow assessing effects on blood pressure and duration of action in healthy volunteers.

A different aspect of applying investigational drugs to healthy volunteers or patients is the safety of study participants. In healthy volunteers, adverse events have been shown to be common and to occur with a frequency of about 10% (Sibille et al. 1998; Lutfullin et al. 2005). However, the majority of adverse events are of mild to moderate intensity (Sibille et al. 1998; Lutfullin et al. 2005). Pursuing Maximum Tolerated Dose concepts (Sibille et al. 1998) appears to be associated with a somewhat higher rate of medically worrying events than concentration or biomarker guided approaches (Lutfullin et al. 2005). As for desired effects as blood pressure reduction, patients are believed to be more sensitive for the occurrence of undesired effects. In the present studies there was, however, no major difference in the quantity and quality of Adverse Events in patients and in healthy volunteers. One patient had to prematurely stop dosing because of an orthostatic hypotension. However, there was also one healthy volunteer who also showed this adverse event, although under both active drug and placebo treatment. Thus, from the point of view of safety and tolerability, PoM or PoC studies can be performed in healthy volunteers or mildly diseased patients without compromising the safety of the study participants.

In conclusion, single dosing in healthy volunteers is sufficient for an early go/no-go decision for the Angiotensin-II receptor antagonist by providing PoM by use of a laboratory biomarker and allows establishing a dose-effect relationship predictive for short-term effects in patients. PoC can be achieved either with single dosing in patients with hypertension or a provocation study with Angiotensin II in healthy volunteers. The potency of the compound, the duration of action and the time to steady state in blood pressure reduction may only be fully assessed following longer treatment periods.

## Figures and Tables

**Table 1:** Study and subject characteristics of studies in healthy volunteers and patients with essential hypertension.

Dose	Design	Medication	Planned subjects	Enrolled subjects	Age	Weight
Healthy volunteers			active treatment/placebo	active treatment/placebo	years mean ± sd	kg mean ± sd
10 mg	SD, CO	Solution	6	5	33.9 ± 5.4	78 ± 9.0
20 mg	SD, CO	Solution	12	12	32.0 ± 5.8	79 ± 12.4
40 mg	SD, CO	Solution	12	12	34.2 ± 5.1	83.5 ± 12.7
80 mg	SD, CO	Solution	12	10	29.8 ± 6.1	71.9 ± 9.6
200 mg	SD, CO	Capsule	12	9	30.4 ± 5.7	76.3 ± 6.4
300 mg	SD, CO	Capsule	12	12	31.8 ± 5.2	75.8 ± 7.8

Healthy volunteers						
50 mg	MD, PG	Capsule	6/2	6/2	31 ± 5.7	78.1 ± 14.4
100 mg	MD, PG	Capsule	6/2	6/2	32.3 ± 6.2	81.8 ± 14.2
200 mg	MD, CO	Capsule	12	9	30.4 ± 5.7	76.3 ± 6.4
300 mg	MD, CO	Capsule	12	10	34.2 ± 6.8	76.5 ± 10.0

Hypertensive Patients						
20 mg	MD, PG	Capsule	10/2	10/2	56.1 ± 8.4	93.7 ± 13.2
50 mg	MD, PG	Capsule	10/2	10/2	58.0 ± 6.6	89.9 ± 16.7
100 mg	MD, PG	Capsule	10/2	9/2	53.0 ± 7.1	80.5 ± 12.4
200 mg	MD, PG	Capsule	10/2	9/2	51.9 ± 9.9	81.4 ± 9.3
300 mg	MD, PG	Capsule	10/2	10/2	53.9 ± 8.9	82.2 ± 9.8
400 mg	MD, PG	Capsule	10/2	9/2	52.8 ± 9.6	87.0 ± 13.1

SD = single dose; MD = multiple dose; CO = crossover; PG = parallel group.

**Table 2:** Baseline values and maximum increase in Renin and Angiotensin following sd administration of BAY 10-6734 to healthy volunteers.

Dose [mg/day]	Time	Healthy volunteer	
		Renin [μg/l/h] mean ± sd	Angiotensin [ng/l] mean ± sd
10 mg	Baseline	0.36 ± 0.20	16.5 ± 07.0
	Peak day 1	1.10 ± 0.62*	20.4 ± 9.3*
20 mg	Baseline	0.55 ± 0.51	15.3 ± 5.3
	Peak day 1	2.89 ± 5.25	23.6 ± 7.7*
40 mg	Baseline	0.29 ± 0.13	4.1 ± 3.2
	Peak day 1	0.40 ± 0.24	9.7± 3.8***
80 mg	Baseline	0.50 ± 0.33	6.4 ± 3.3
	Peak day 1	3.26 ± 2.92**	13.7 ± 4.7***
200 mg	Baseline	0.51 ± 0.35	8.6 ± 8.3
	Peak day 1	7.55 ± 6.75**	46.1 ± 43.8*
300 mg	Baseline	0.47 ± 0.57	8.8 ± 3.9
	Peak day 1	8.96 ± 4.39***	58.4 ± 32.4***

* p < 0.05

** p < 0.01

*** p < 0.001

**Table 3:** Baseline values and maximum increase in Renin and Angiotensin following md administration of BAY 10-6734 for 7 days to healthy volunteers and patients with essential hypertension.

Dose [mg/day]	Time	Healthy volunteer		Patient	
		Renin [μg/l/h] mean ± sd	Angiotensin [ng/l] mean ± sd	Renin [μU/ml] mean ± sd	Angiotensin [ng/l] mean ± sd
20 mg	Baseline	n.a.	n.a.	18.5 ± 7.9	9.3 ± 1.9
	Peak day 1	n.a.	n.a.	37.6 ± 20.0*	13.9 ± 4.1***
	Peak day 7	n.a.	n.a.	41.3 ± 20.9	14.0 ± 4.3***
50 mg	Baseline	0.89 ± 01.48	019.0 ± 11.9	11.8 ± 3.2	9.5 ± 1.3
	Peak day 1	4.56 ± 7.28**	58.4 ± 29.6*	30.6 ± 12.0**	14.7 ± 3.2**
	Peak day 7	13.11 ± 23.75**	147.7 ± 85.6*	37.8 ± 15.92*	20.5 ± 18.0
100 mg	Baseline	0.37 ± 0.25	4.2 ± 4.4	17.5 ± 13.9	16.6 ± 5.8
	Peak day 1	3.10 ± 3.22	10.0 ± 4.8	60.8 ± 68.3	39.6 ± 36.7
	Peak day 7	8.35 ± 10.30	47.7 ± 38.8*	139.4 ± 188.3	114.9 ± 34.9
200 mg	Baseline	0.38 ± 0.20	7.1 ± 4.4	14.6 ± 5.6	12.8 ± 3.1
	Peak day 1	4.00 ± 3.59*	42.9 ± 32.8*	46.9 ± 26.7*	23.5 ± 3.0**
	Peak day 7	25.21 ± 8.57***	196.2 ± 140.5*	107.1 ± 79.8*	43.8 ± 28.1*
300 mg	Baseline	0.60 ± 0.44	5.6 ± 2.3	18.5 ± 5.8	21.0 ± 7.5
	Peak day 1	3.06 ± 2.16**	18.8 ± 15.2*	82.3 ± 62.1*	47.2 ± 24.8
	Peak day 7	18.19 ± 9.71***	130.0 ± 83.9***	156.6 ± 128.8*	82.0 ± 57.1*
400 mg	Baseline	n.a.	n.a.	20.9 ± 14.0	20.7 ± 10.8
	Peak day 1	n.a.	n.a.	102.5 ± 114.2*	43.8 ± 41.9
	Peak day 7	n.a.	n.a.	132.9 ± 161.1*	39.6 ± 26.9*

* p < 0.05 compared to baseline

** p < 0.01 compared to baseline

*** p < 0.001 compared to baseline

n.a. = not applicable

**Table 4:** Diastolic blood pressure (mean +/− SD) in patients with essential hypertension at screening, at baseline (mean of three measurements) and 12 and 24 hours after administration of the last dose of BAY 10-6734.

Dose [mg/day]	Diastolic Blood Pressure in Hypertensive Patients (mm Hg)			
	Screening	Baseline	7 days 12 hours	7 days 24 hours
20 mg	98,8 + 9,6	103,3 + 6,7	92,0 + 8.8	97,6 + 6,2
50 mg	97,8 ± 8,5	105,6 ± 5,1	88,6 ± 10,9	94,8 ± 5,3
100 mg	100,4 ± 10,2	104,7 ± 6,7	92,0 ± 7,7	83,6 ± 6,1
200 mg	98,6 ± 9,4	99,7 ± 4,0	88,6 ± 7,9	90,6 ± 6,8
300 mg	98,9 ± 6,9	101,0 ± 5,2	86,6 ± 9,6	93,6 ± 6,8
400 mg	100,7 ± 6,6	103,2 ± 4,3	85,6 ± 7,2	92,2 ± 7,5

**Table 5:** Pharmacokinetic parameters of BAY 10-6734 and BAY 10-6735 following sd administration to healthy volunteers (geom. mean +− geom. sd)

	10 mg		20 mg		40 mg		80 mg		200 mg		300 mg	
	BAY	BAY	BAY	BAY	BAY	BAY	BAY	BAY	BAY	BAY	BAY	BAY
						
	10-6734	10-6735	10-6734	10-6735	10-6734	10-6735	10-6734	10-6735	10-6734	10-6735	10-6734	10-6735
AUC	25,39	755,8	30,74	1295,7	73,2	2619,9	169,7	5651,4	366,4	14,495	520,1	27281,6
μg × h/l	1,52	1,4	1,4	1,1	1,4	1,3	1,3	1,4	1,8	1,4	1,5	1,5
AUC_norm_	196,7	6037,4	119,9	5211,5	154,1	5688,2	152,8	5247,9	147,03	5996,7	135,7	7337
kg × h/l	1,47	1,4	1,5	1,2	1,3	1,3	1,2	1,4	1,7	1,4	1,5	1,5
C_max_	54,8	237,2	44,2	462,9	92,8	994,8	202,7	2477,7	378,3	4923,9	416,9	8373,9
μg/l	1,64	1,4	1,6	1,2	1,6	1,2	1,5	1,4	1,7	1,47	1,5	1,4
C_max/norm_	442,5	1894,5	172,5	1861,8	195,5	2159,7	182,6	2300,8	151,8	2037	108,7	2252,1
kg/l	1,6	1,4	1,7	1,3	1,4	1,2	1,5	1,3	1,6	1,5	1,4	1,4
T_1/2_	0,29	5,7	0,39	4,26	0,33	4,77	0,26	7,03	0,61	5,22	0,93	6,39
h	1,31	1,3	1,42	1,24	1,5	1,1	1,5	1,2	1,4	1,2	1,9	1,5

AUC = area under the curve

C_max_ = maximum plasma concentration

T_1/2_ = terminal half-life
